# Supplementary material for: New insights into the evolution of SPX gene family from algae to legumes; a focus on soybean
Source: BMC Genomics. 2021 Dec 30;22:915. doi: 10.1186/s12864-021-08242-5 (PMC8717665; doi:10.1186/s12864-021-08242-5)
Supplement: Supplementary file 1 — Additional file 1. [file 12864_2021_8242_MOESM1_ESM.pdf]

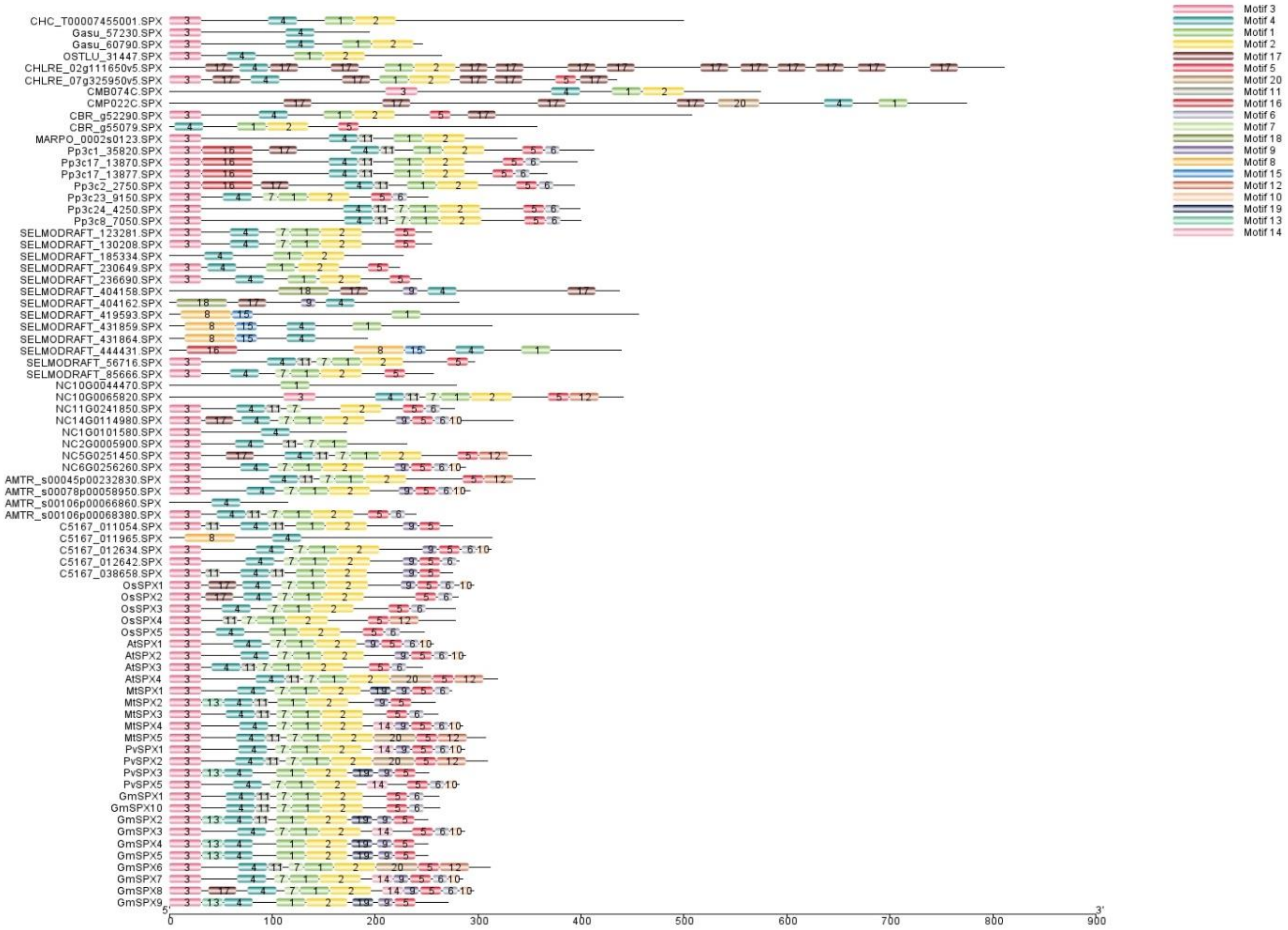

Figure S1. Motif loss and gain in SPX class genes during the evolution from algae to current Angiosperms

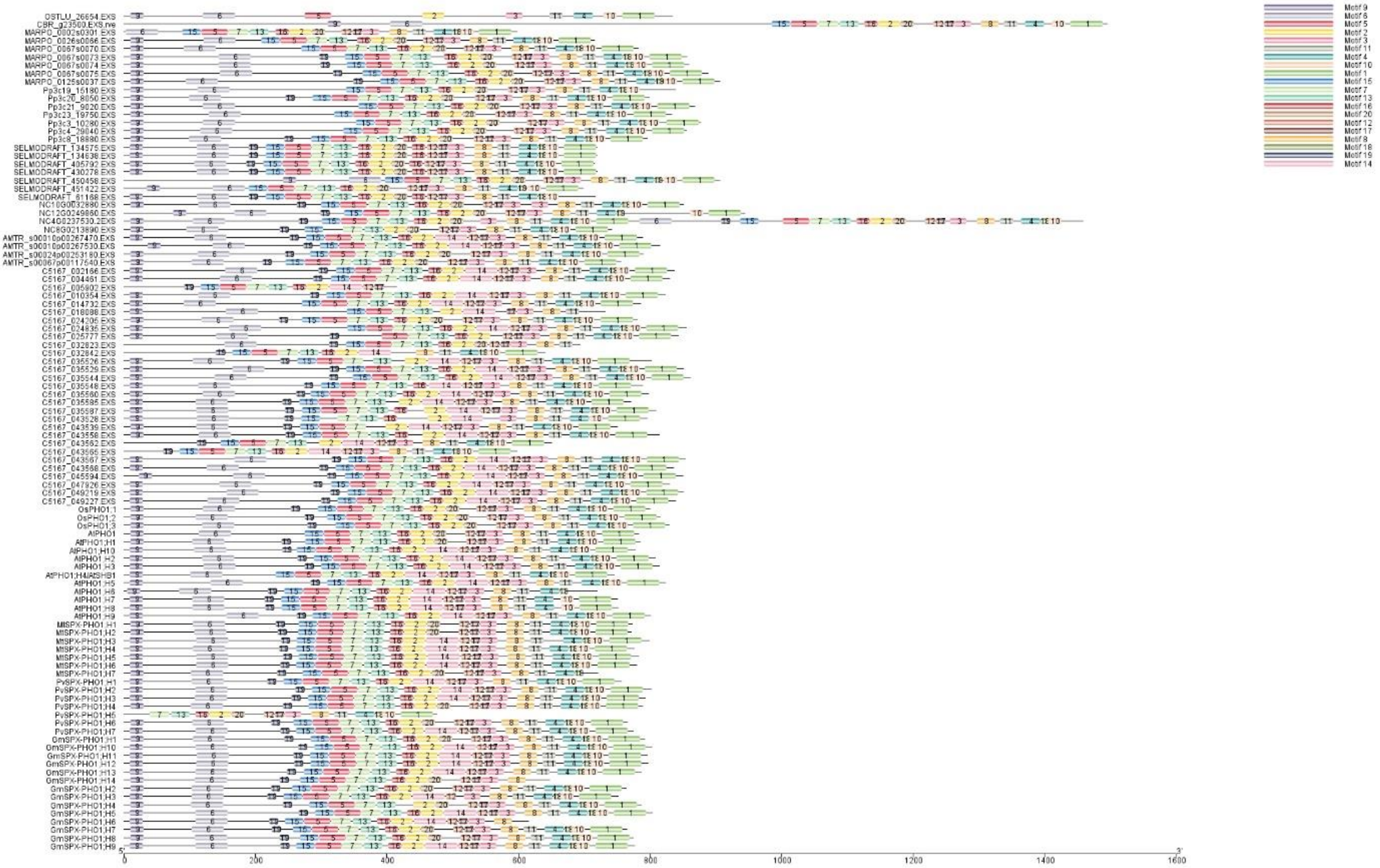

Figure S2. Motif loss and gain in SPX-EXS class genes during the evolution from algae to current Angiosperms

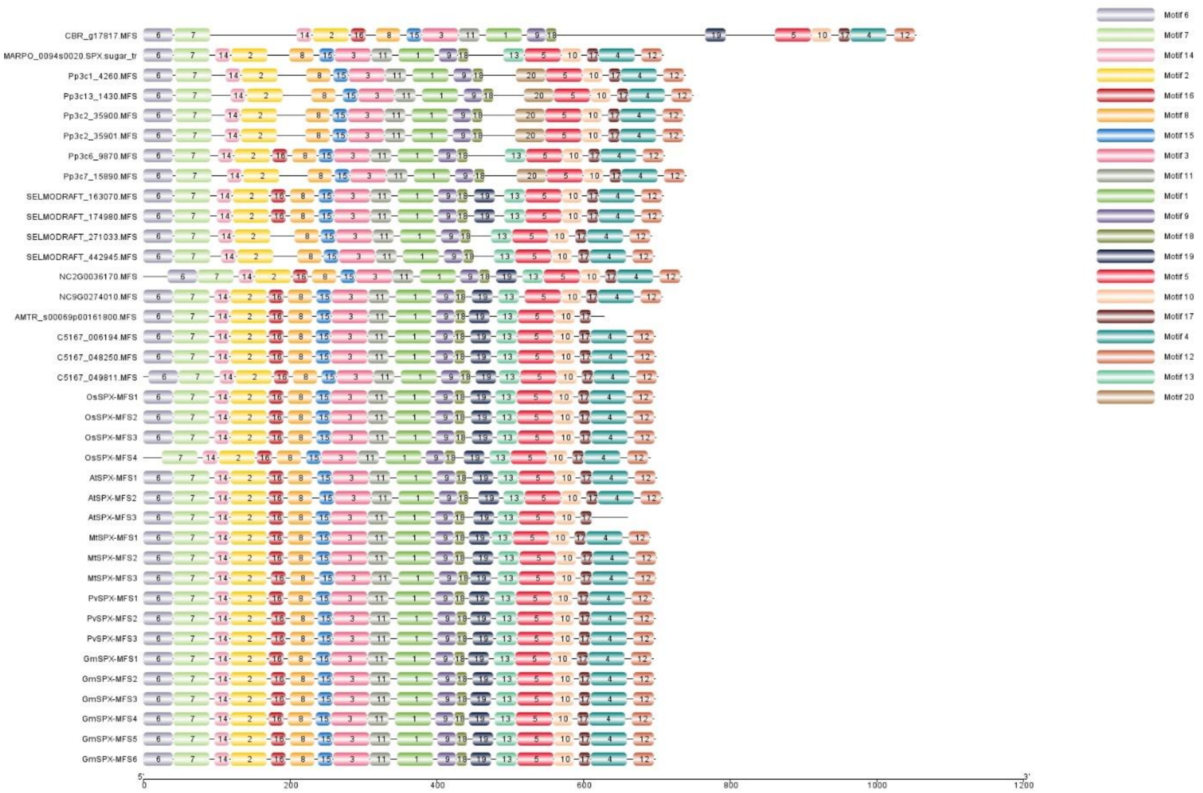

Figure S3. Motif loss and gain in SPX-MFS class genes during the evolution from algae to current Angiosperms

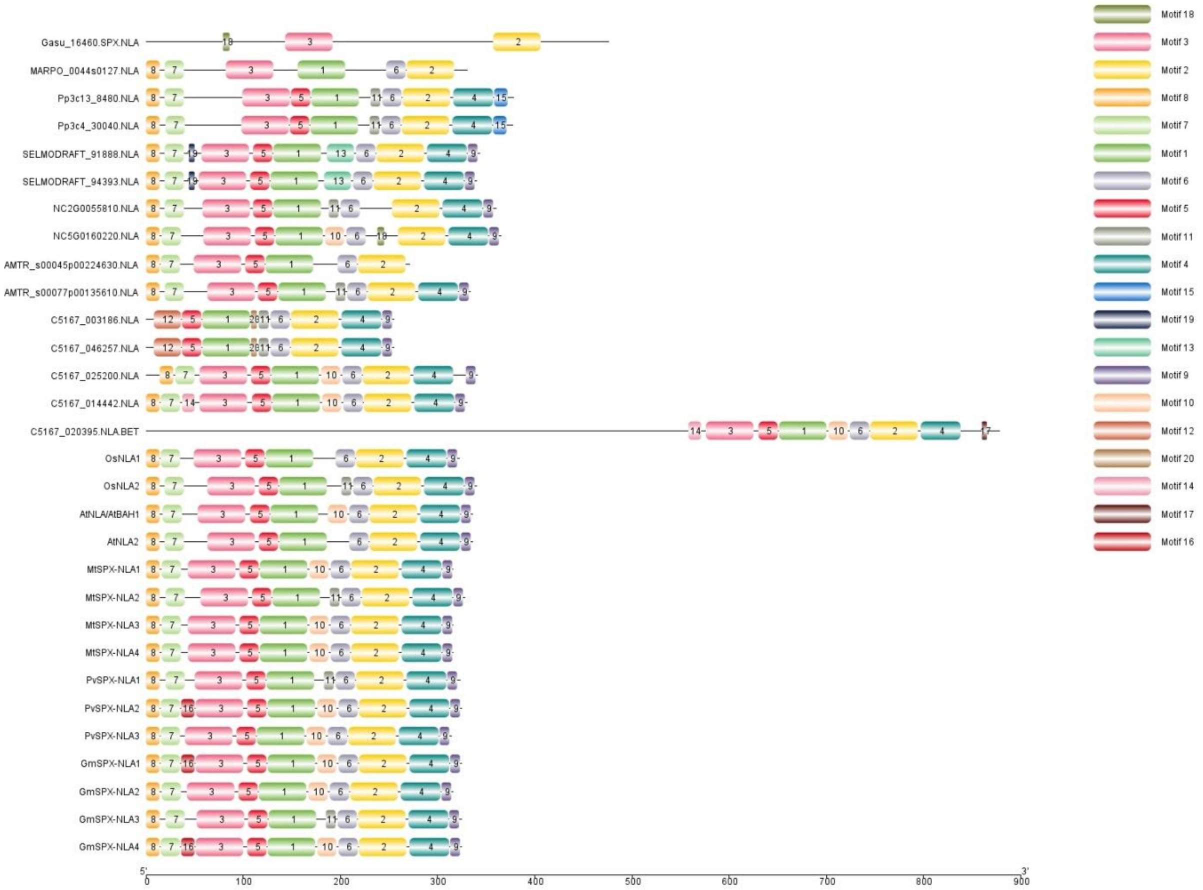

Figure S4. Motif loss and gain in SPX-RING class genes during the evolution from algae to current Angiosperms

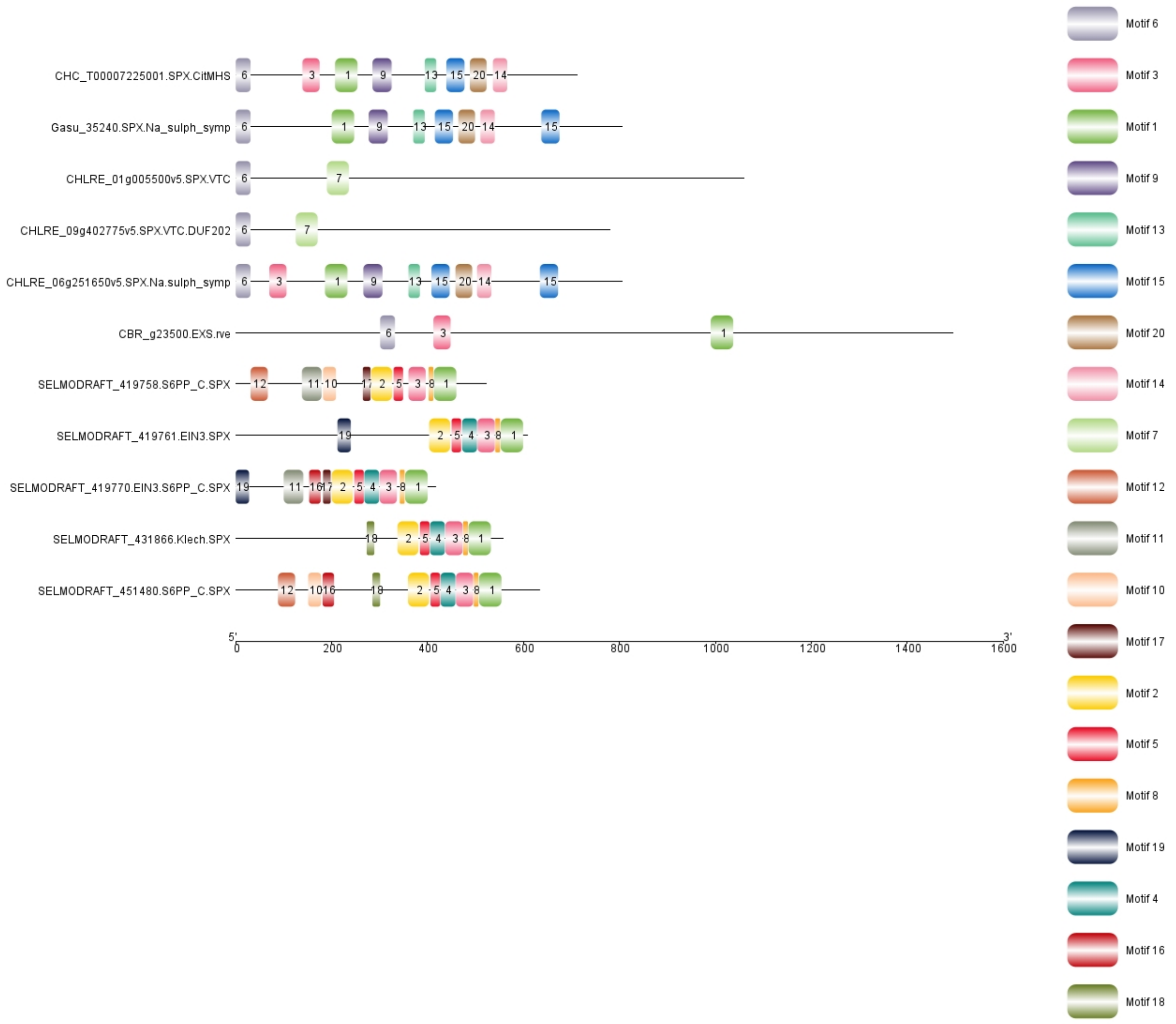

Figure S5. Motifs specifically-found in the new classes of SPX proteins in basal plants

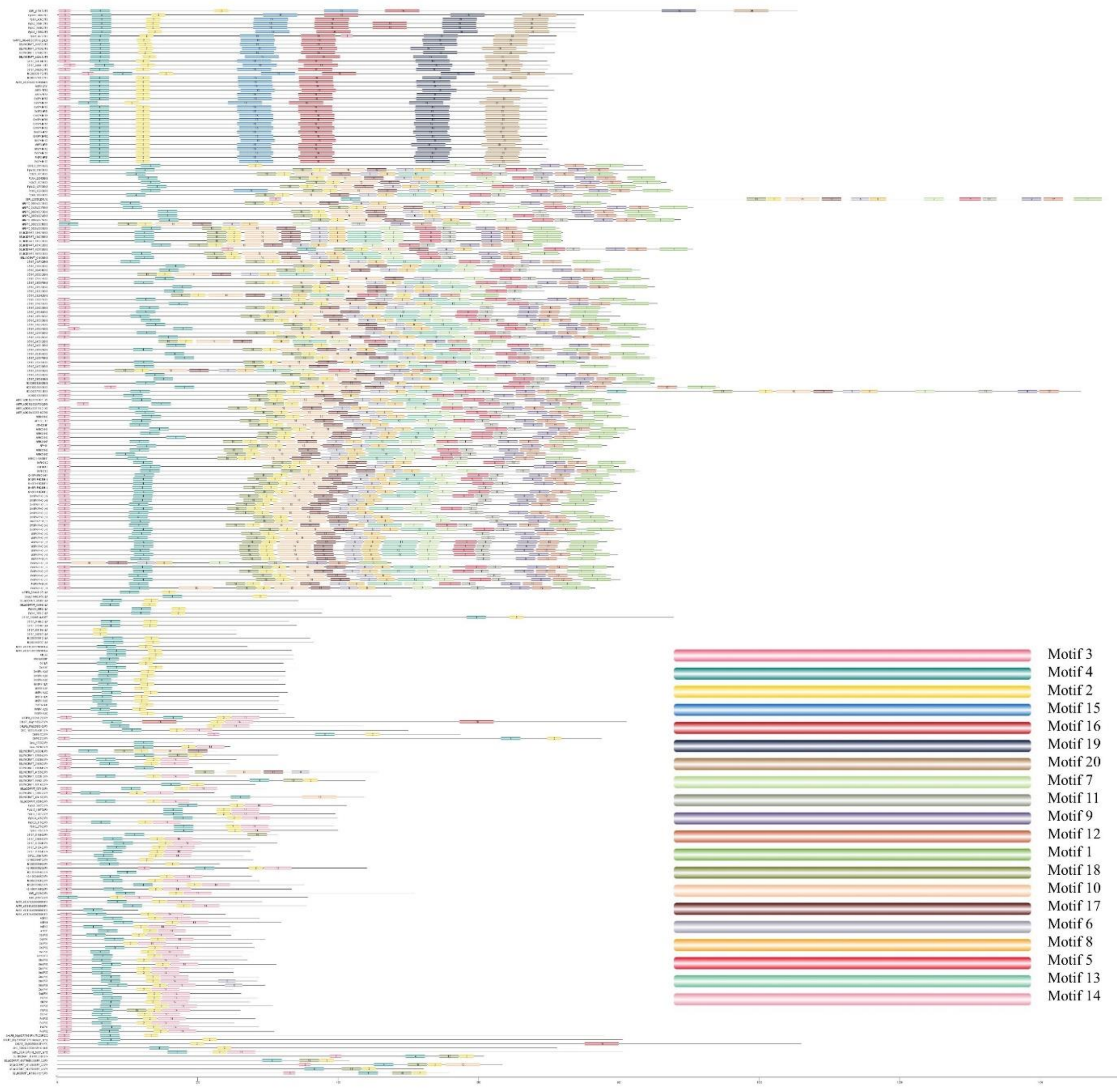

Figure S6. Motif loss and gain of all SPX proteins during the evolution from algae to current Angiosperms

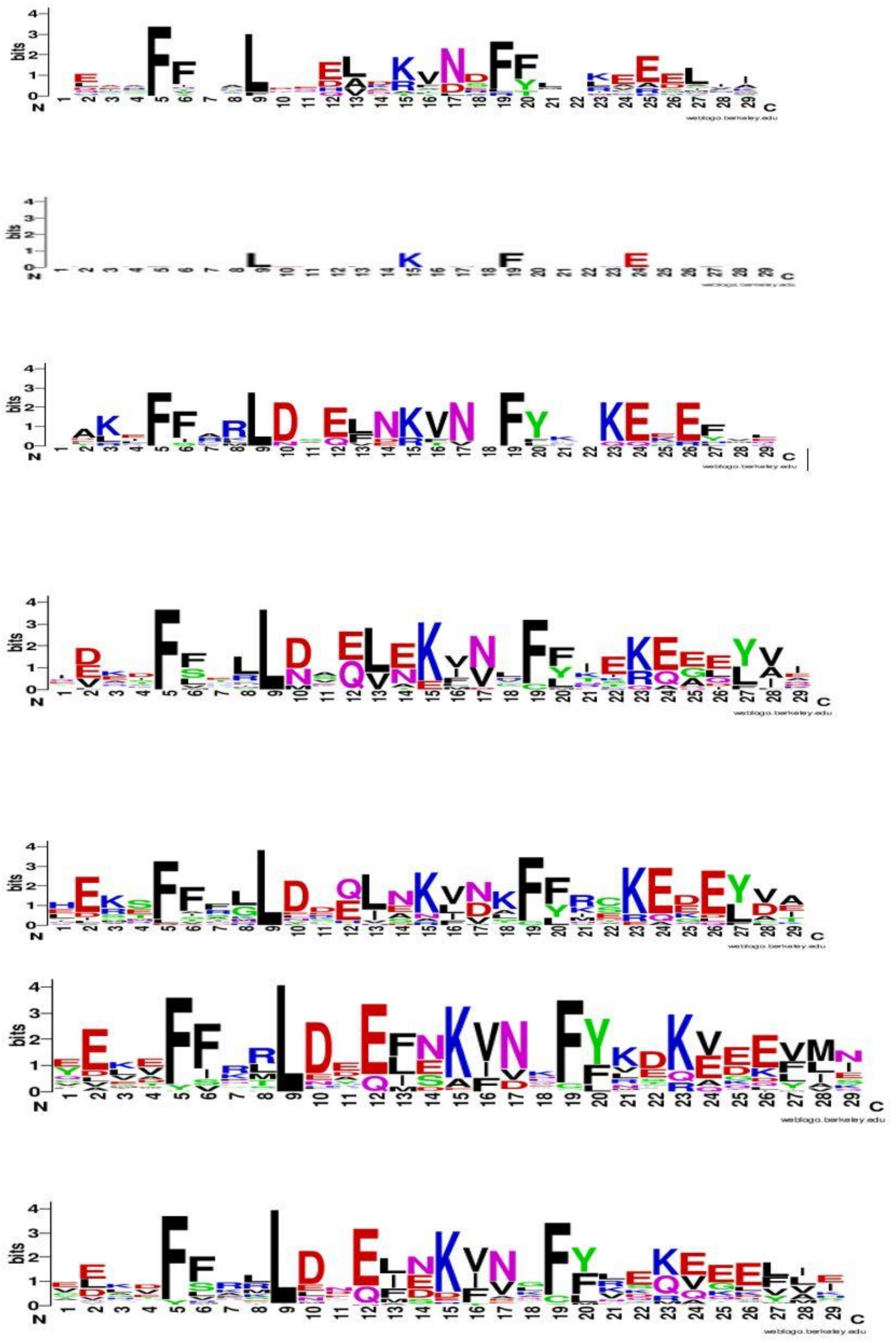

Figure S7. Consensus sequences of motif 4 in SPX domain conserved in whole SPX proteins; in different phyla. Order of phyla from up to down: algae (*C.reinhardttii*, *O. lucimarinus*, *G. sulfuraria*, C. *crispus*, *C. merolae*), charophytes (*C. braunii*), liverwort (*M. polymorpha*), bryophytes (*P. patens*), lycophytes (*S. moellendorffii*), basal angiosperms (*A. thricopoda*, *P. sumniferum*, *N. colorata*), and current angiosperm (Arabidopsis, rice, soybean, common bean, alfalfa).

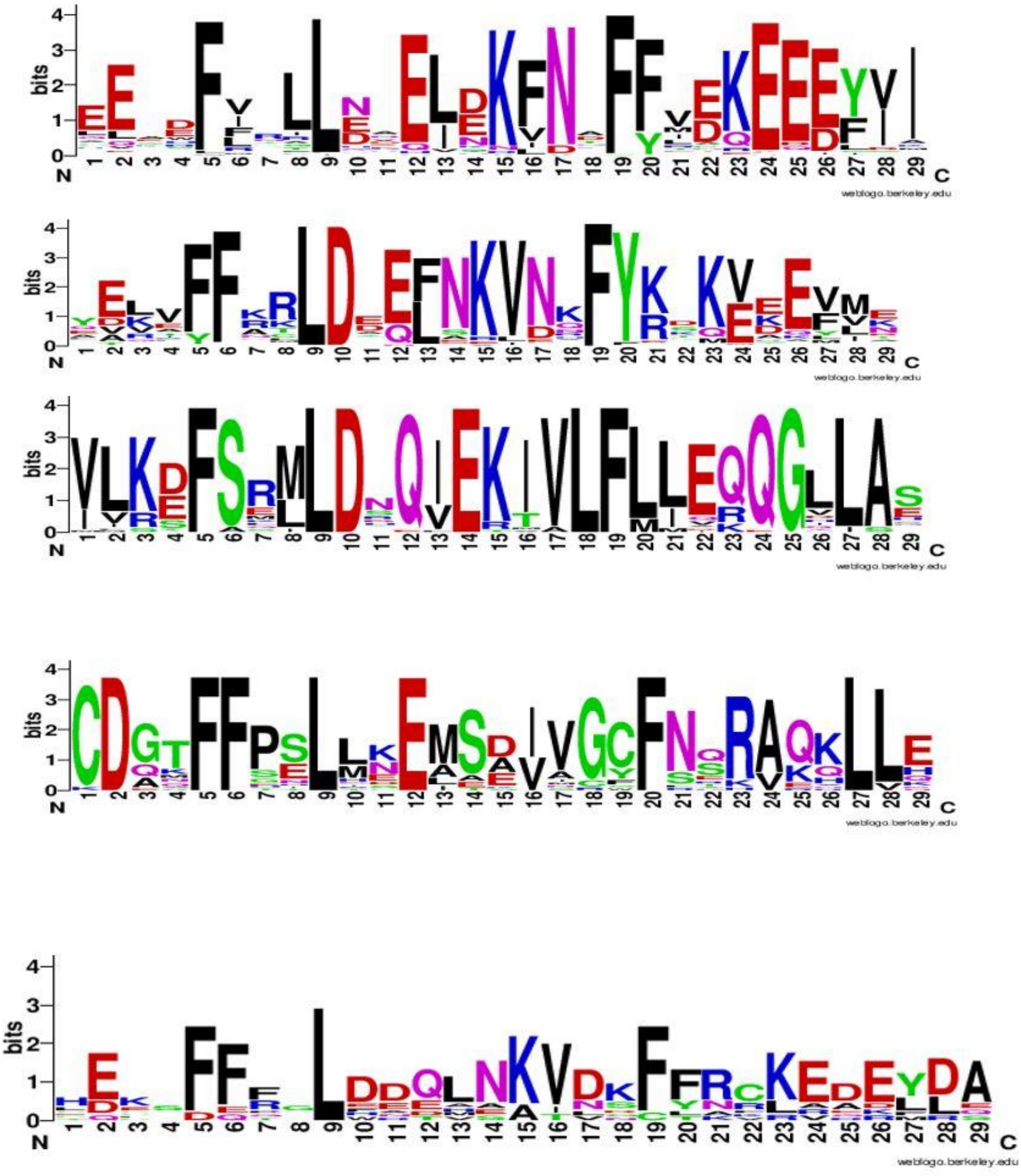

Figure S8. Consensus sequences of motif 4 in SPX domain conserved in whole SPX proteins; in different classes. Order of different classes from up to down: SPX, EXS, MFS, RING, new identified classes.

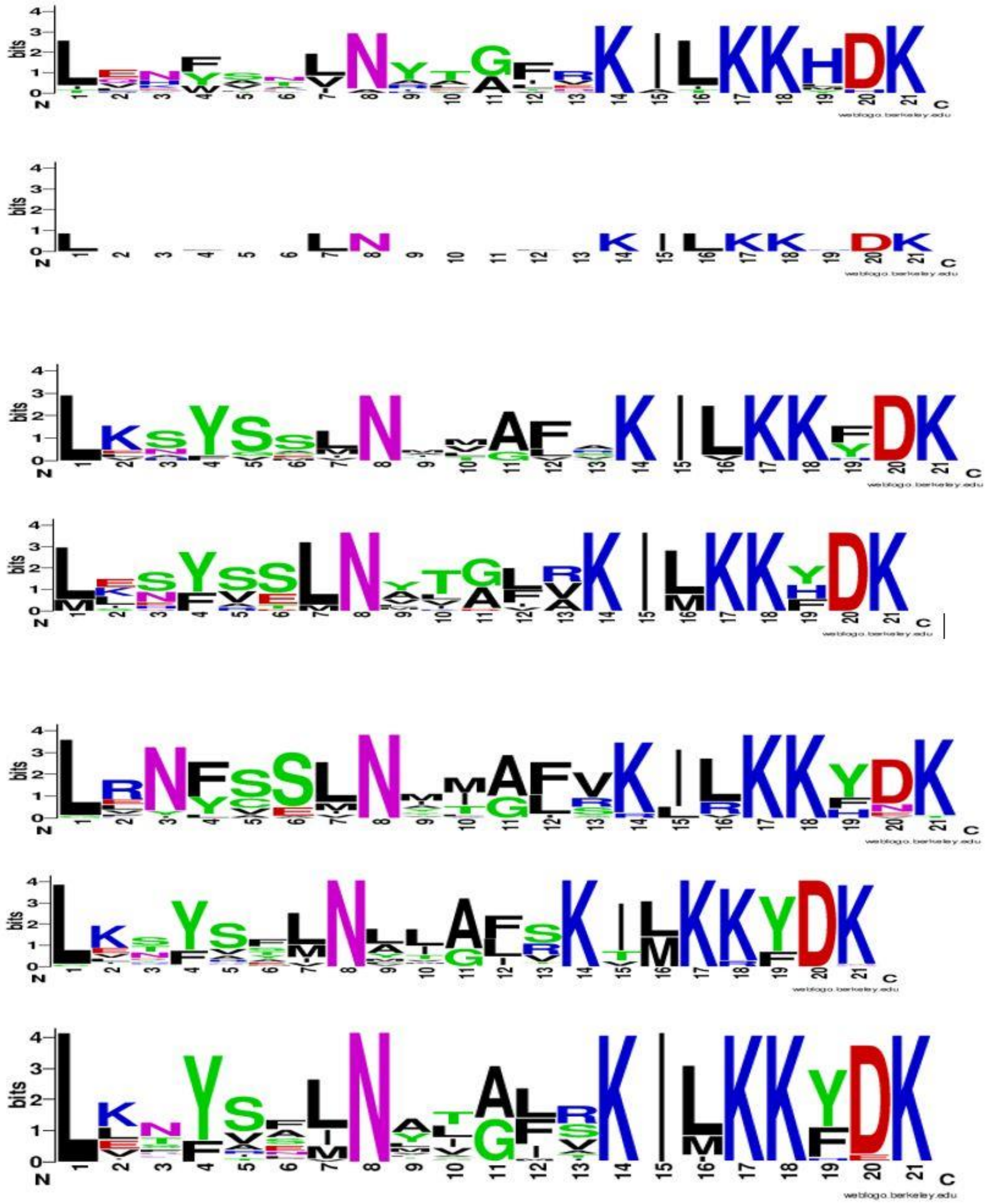

Figure S9. Consensus sequences of motif 2 in SPX domain conserved in whole SPX proteins; in different phyla. Order of phyla from up to down: algae (*C.reinhardttii*, *O. lucimarinus*, *G. sulfuraria*, C. *crispus*, *C. merolae*), charophytes (*C. braunii*), liverwort (*M. polymorpha*), bryophytes (*P. patens*), lycophytes (*S. moellendorffii*), basal angiosperms (*A. thricopoda*, *P. sumniferum*, *N. colorata*), and current angiosperm (Arabidopsis, rice, soybean, common bean, alfalfa).

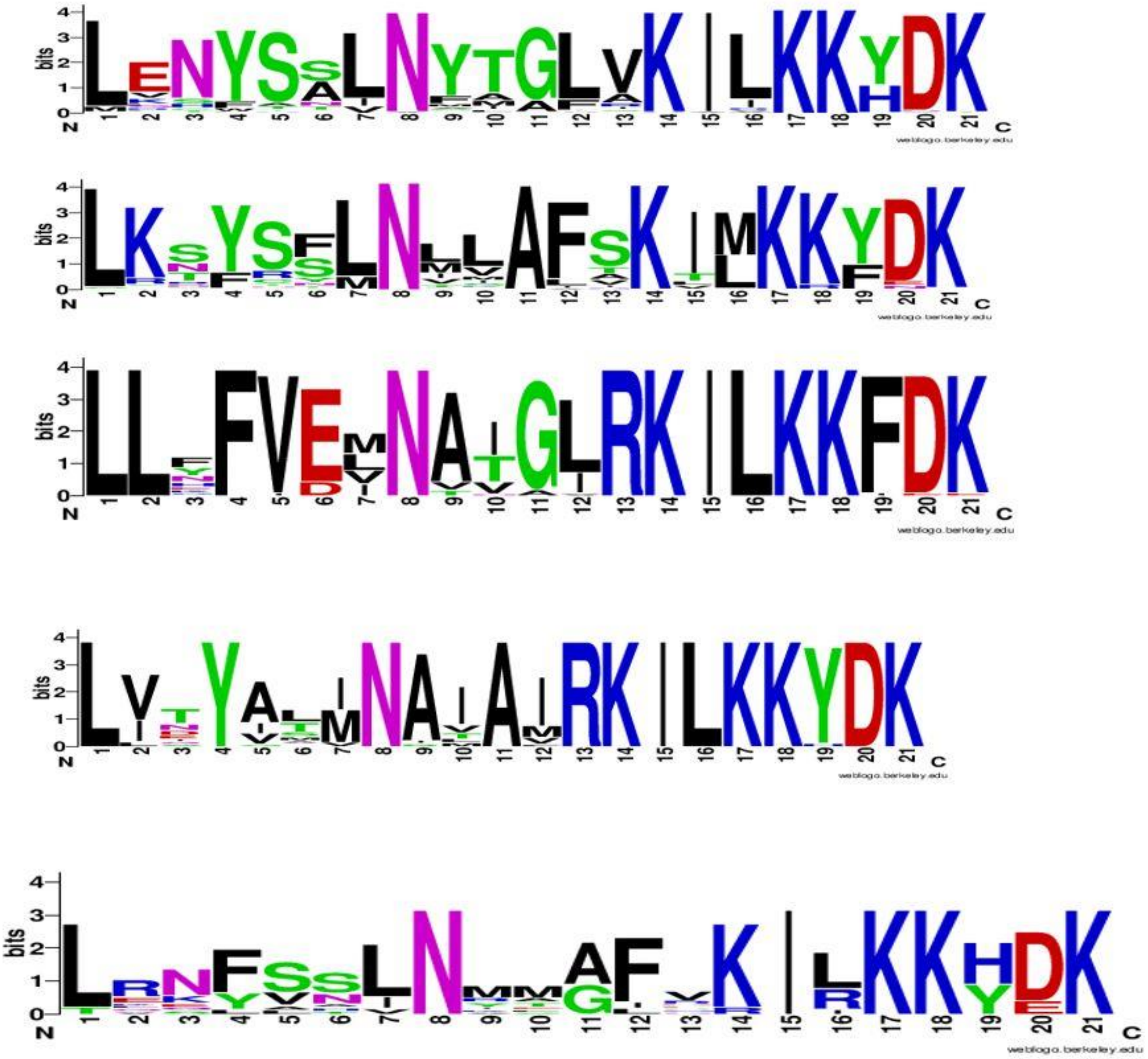

Figure S10. Consensus sequences of motif 2 in SPX domain conserved in whole SPX proteins; in different classes. Order of different classes from up to down: SPX, EXS, MFS, RING, new identified classes.

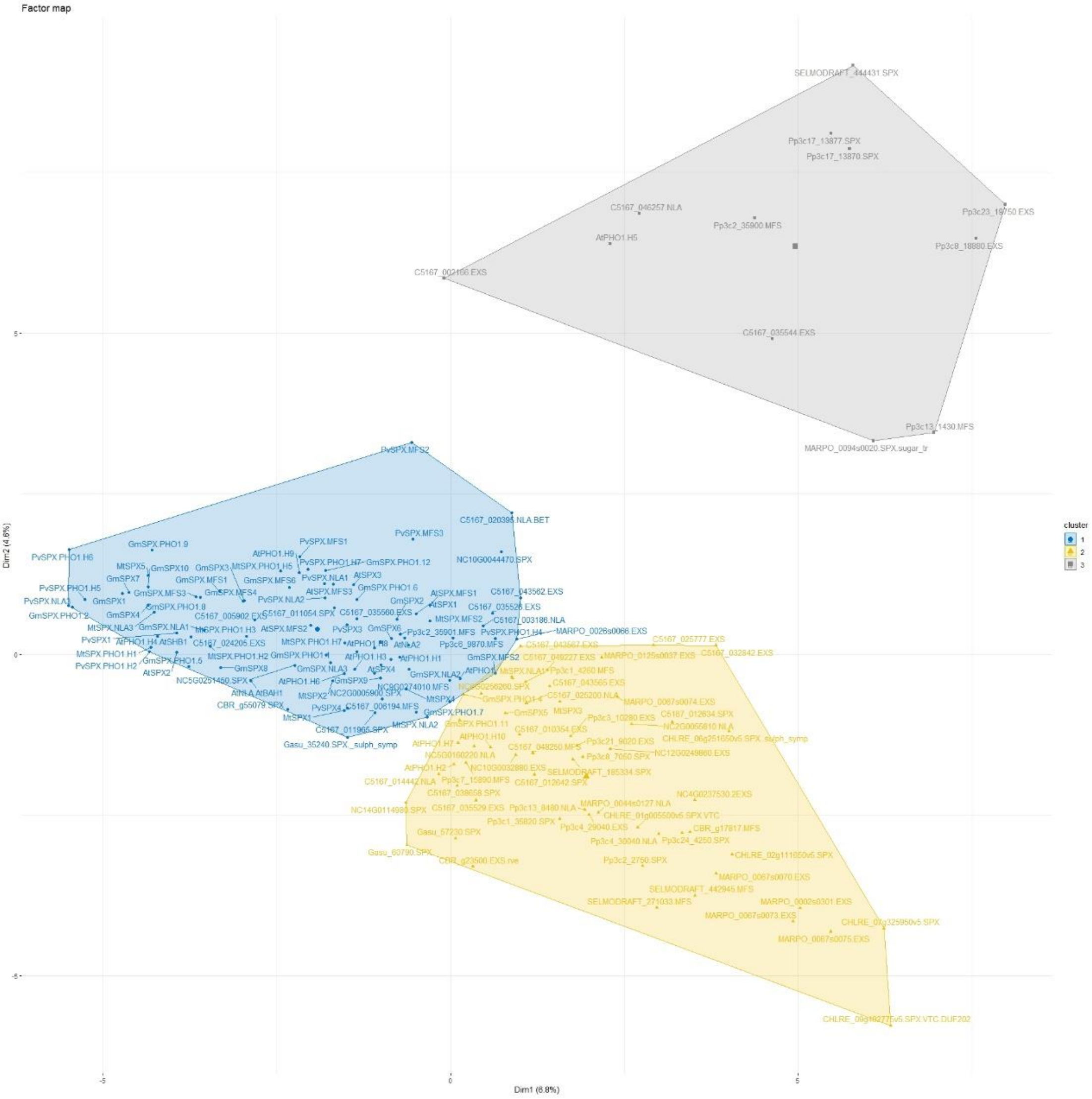

Figure S11. Hierarchical Clustering on Principal Components (HCPC) of SPXs in the lower plants and current Angiosperms based on presence or absence of Cis-acting elements in their promoters.

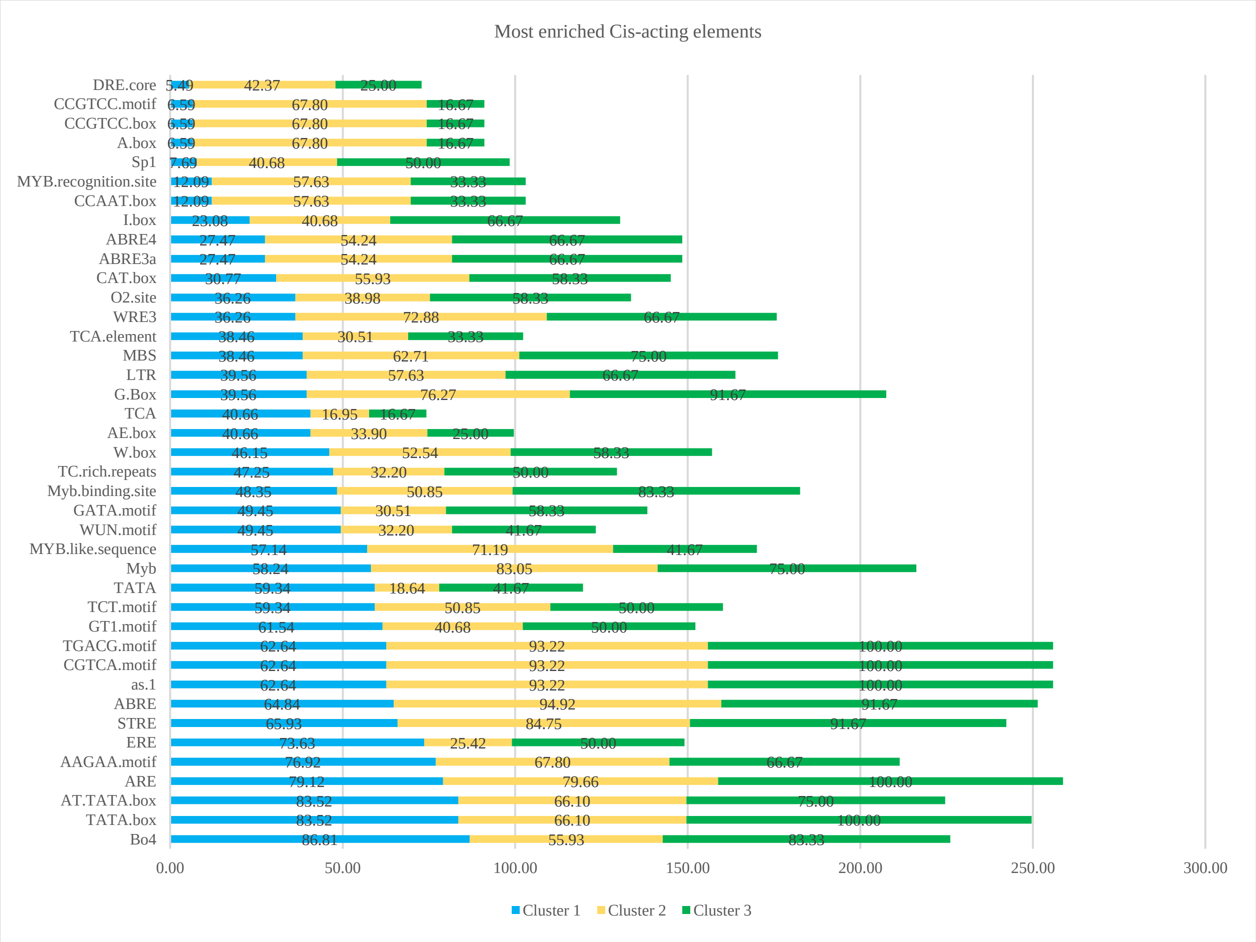

Figure S12. Production of genes in each cluster containing the most frequent Cis-acting elements. The clusters were shown in different colors: cluster 1= blue, cluster 2= yellow, and cluster 3= green.

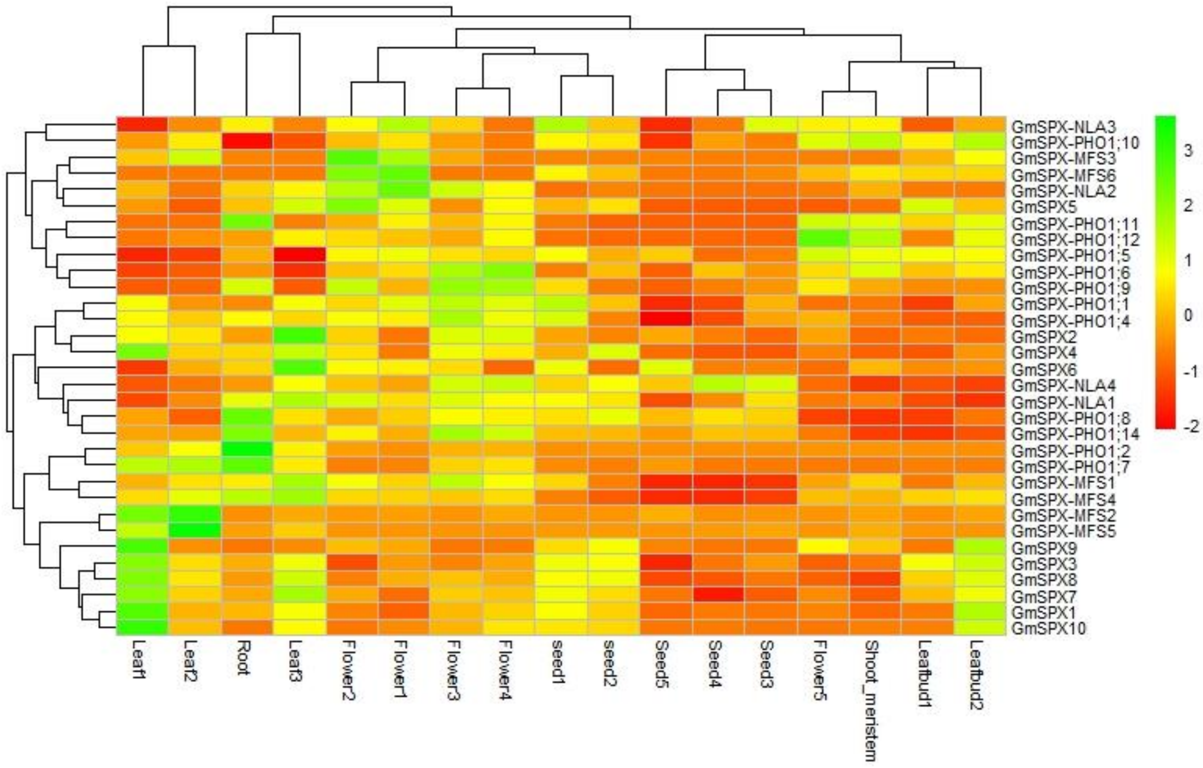

Figure S13. Expression levels of *GmSPXs* in the different developmental stages of different tissues. Using data from PRJNA238493 bioproject.

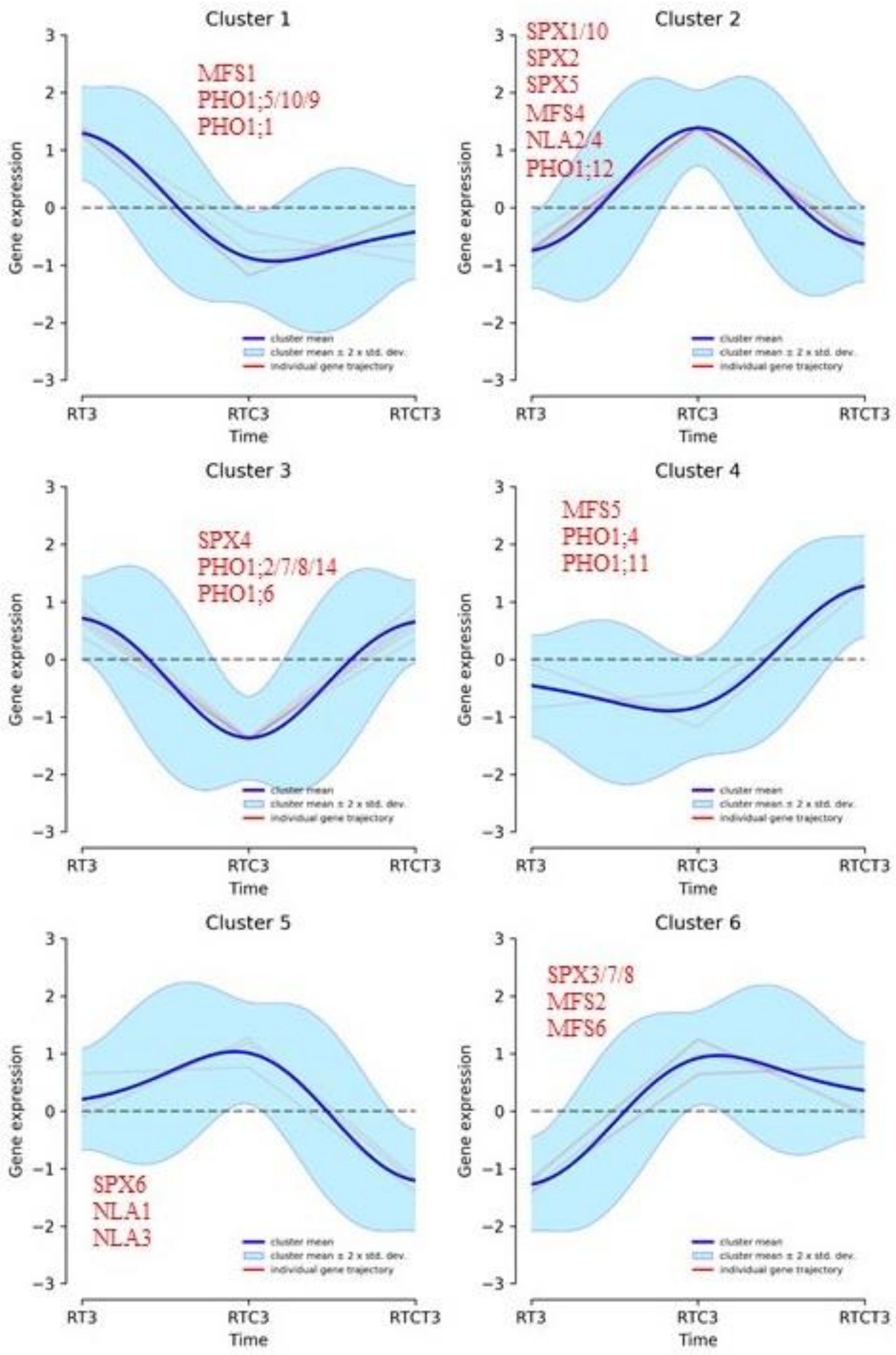

Figure S14. Regulation of SPX genes by phosphate starvation in the roots. DPGP analysis was performed for expression pattern of GmSPXs in roots during three time-points; RT= P deficiency, RTC= P deficiency and recovery, and RTCT = P deficiency, recovery, and second P deficiency. Shown are clustered trajectories of GmSPX genes. The cluster means are in blue, the individual SPX genes are shown in red. Using data from PRJNA544698 bioproject.

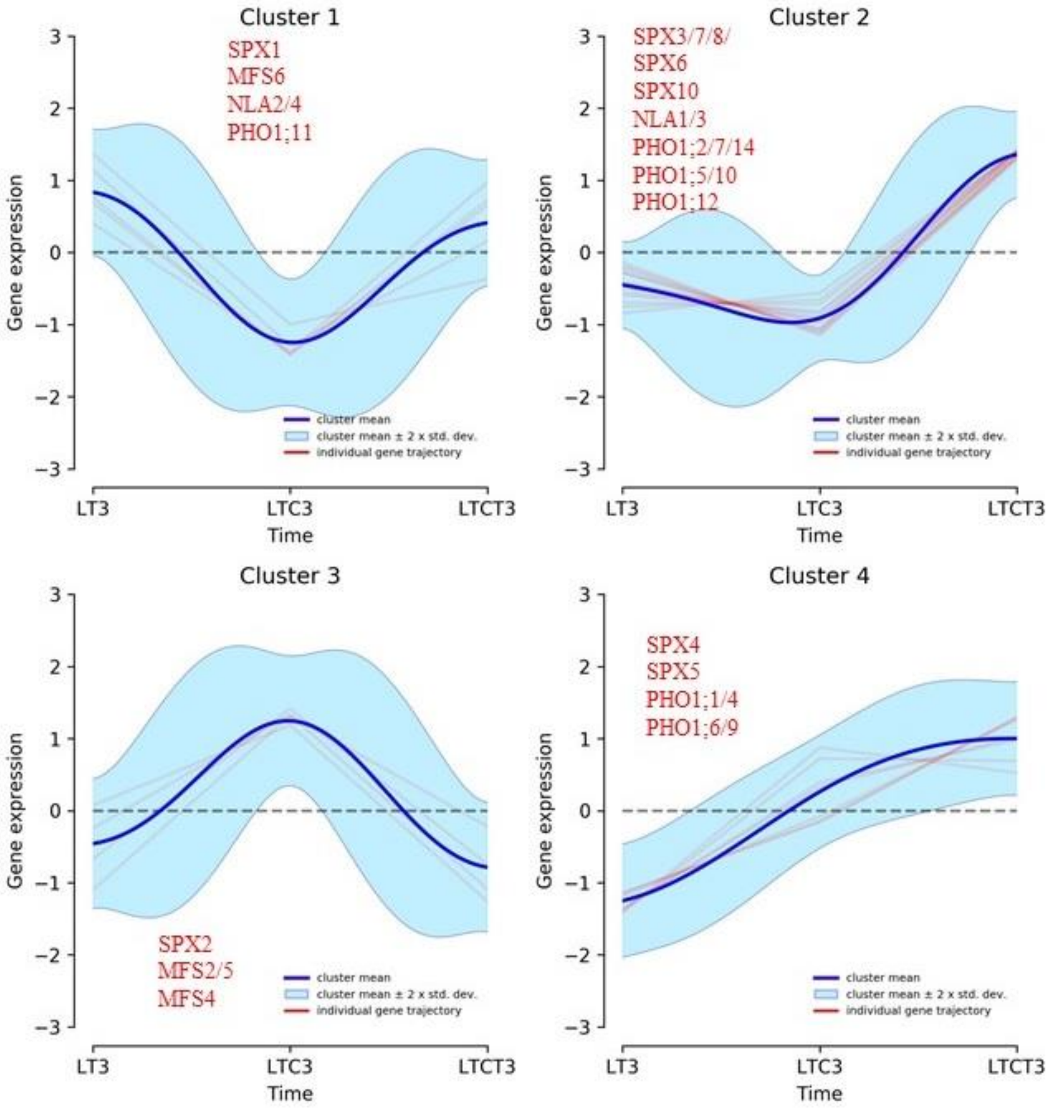

Figure S15. Regulation of SPX genes by phosphate starvation in the leaves. DPGP analysis was performed for expression pattern of GmSPXs in leaves during three time-points; RT= P deficiency, RTC= P deficiency and recovery, and RTCT = P deficiency, recovery, and second P deficiency. Shown are clustered trajectories of GmSPX genes. The cluster means are in blue, the individual SPX genes are shown in red. Using data from PRJNA544698 bioproject.

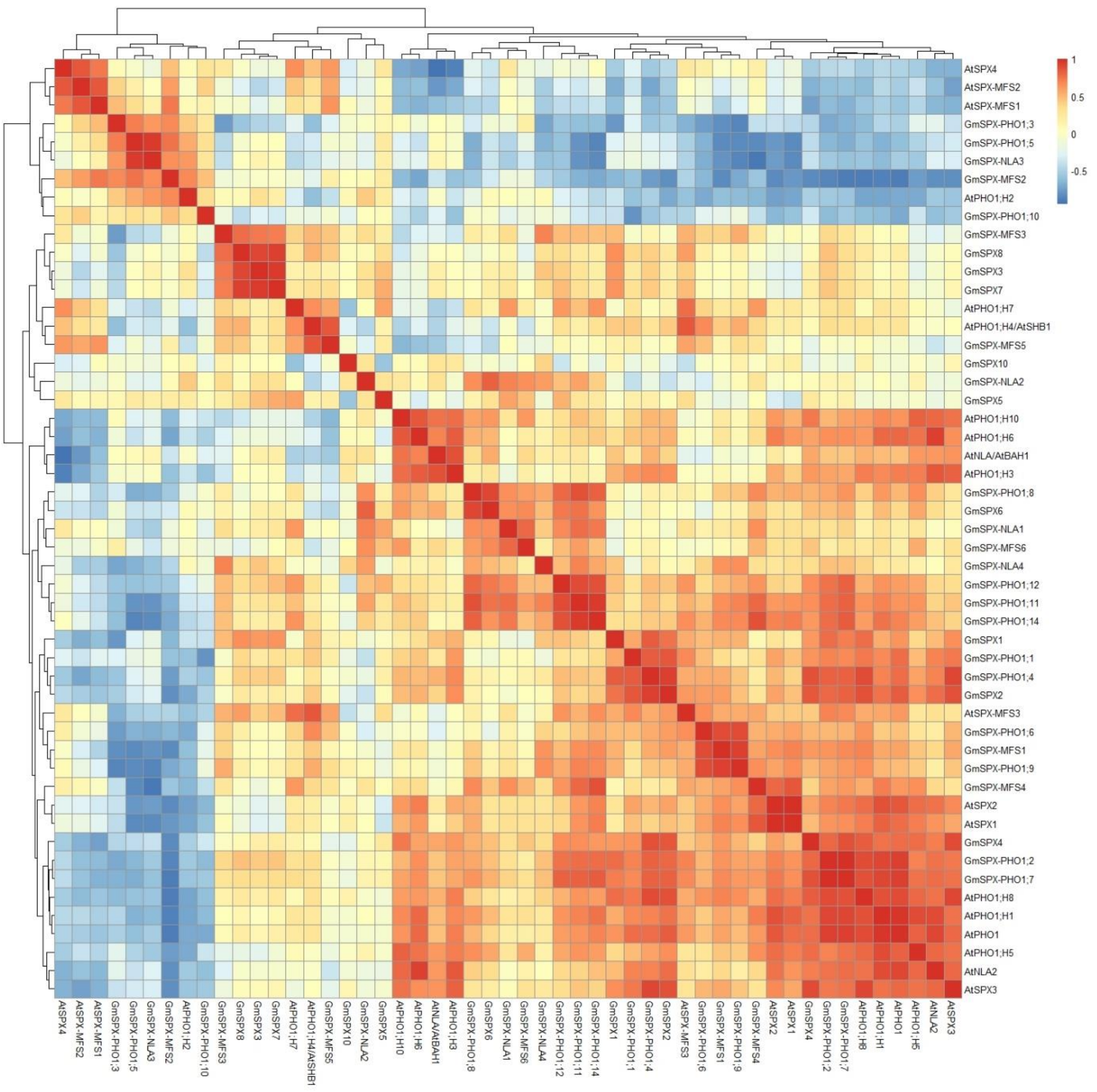

Figure S16. Correlation heat map of SPX genes in soybean and Arabidopsis using RNA-seq datasets from three different zones of roots (GSE64665).
