# Supplementary material for: New insights into the evolution of SPX gene family from algae to legumes; a focus on soybean
Source: BMC Genomics. 2021 Dec 30;22:915. doi: 10.1186/s12864-021-08242-5 (PMC8717665; doi:10.1186/s12864-021-08242-5)
Supplement: Supplementary file 2 — Additional file 2. [file 12864_2021_8242_MOESM2_ESM.pdf]

Table 1. Nutrient solution for soybean growth in N and P deficiency

Chemicals	Concentration	Concentration (deficiency)
Macro elements		
NH4NO3	2.5 mM	0.5 mM
MgSO4.7H2O	500 µM	
KH2PO4	120 µM	20 µM
K2HPO4	30 µM	5 µM
KCl	250 µM	
Fe-Na-EDTA	100 µM	
CaCl2	250 µM	
Trace elements		
H3BO3	46 µM	
MnSO4.H2O	8 µM	
ZnSO4.7H2O	8 µM	
CuSO4.5H2O	2 µM	
Na2MoO4.2H2O		

Table 2. GmSPXs primer sequences used for qRT-PCR

Gene ID	Name	Forward primer sequence	Reverse primer sequence
GLYMA_18G290800	ACT6	AAGCTTTCTTACATTGCCCTTG	CAACTCATAGCTCTTCTCCACA
GLYMA_01G135500	SPX1	CGTTTGCCGTTCATCCAG	TGCCACAGTTATCGCCTC
GLYMA_04G147600	SPX3	CTTGCTCGAAAACTATAGTGCC	AAGCATTGTTTCACACTCCTTC
GLYMA_13G061700	SPX6	AATCGTGGAACTTTCAACTGTG	GTCTTCCAAATGGGTCTTGAAC
GLYMA_13G166800	SPX7	CCTTTGCATAATTTCTCGCGTA	GGTGTTGAATTTGTCGAGTTCA
GLYMA_03G032400	SPX10	TGAAGTTTGGGAAGAGACTCAA	AATACACAAACTCAGTCTCCGT
GLYMA_09G128500	MFS1	CTCATGAAGAAACGAGTGAAGC	GAAATCCTTGAGTACATGTCGC
GLYMA_19G203000	NLA3	TTGGAGTTTATTCTAAGGCCGT	TAGCCTCTCTTTCCAGTAGTCT
GLYMA_02G003700	PHO2	TTCGCTTCATAACCAACAA	GCACTTCATCCTCTTCTG
GLYMA_10G004800	PHO7	CTCACCAAGCACAGCATT	AGAAGACCTACAAAGAACAGAAA
